# The association of HALP score with low muscle mass in older adults

**DOI:** 10.3389/fnut.2025.1618736

**Published:** 2025-08-19

**Authors:** Suxia Liu, Jiansheng Huang, Xiaolei Hu, Shuaiqing Chen, Mingshen Lin

**Affiliations:** ^1^Department of Clinical Laboratory, Lishui Municipal Central Hospital, Fifth Affiliated Hospital of Wenzhou Medical University, Lishui, China; ^2^Department of Cardiology, Lishui Hospital of Traditional Chinese Medicine, Lishui, China

**Keywords:** older adults, low muscle mass, sarcopenia, HALP, mortality, NHANES

## Abstract

**Background:**

The HALP score, combining hemoglobin, albumin, lymphocyte, and platelet parameters, serves as a comprehensive indicator reflecting both inflammatory processes and nutritional conditions. Our investigation aimed to explore the association of this composite score with the prevalence of low muscle mass and associated mortality in the elderly American population.

**Methods:**

The investigation incorporated information from 3,550 individuals aged ≥60 years enrolled in the National Health and Nutrition Examination Survey (NHANES) between 1999 and 2004. Multivariate logistic regression models were employed to assess the presence of low muscle mass, while Cox proportional hazards models examined mortality outcomes. Non-linear associations and inflection points were examined through the application of restricted cubic spline (RCS) methodology. Additional statistical analyses included Kaplan–Meier survival curve, subgroup analyses, interaction testing, and sensitivity analyses.

**Results:**

Participants within the top ln HALP quartile demonstrated a 29% lower probability of having low muscle mass relative to those in the bottom quartile (OR = 0.71, 95% CI: 0.56, 0.89). Participants with low muscle mass in the top quartile of ln HALP had a 23% reduced risk of all-cause mortality compared to those in the bottom quartile (HR = 0.77, 95% CI: 0.62, 0.97). Non-linear modeling using restricted cubic splines established a critical value at ln HALP = 3.9. Below this value, increasing ln HALP was inversely related to both the presence of low muscle mass (OR = 0.56, 95% CI: 0.41, 0.75) and mortality (HR = 0.53, 95% CI: 0.41, 0.68). No meaningful statistical trends were detected beyond this critical value. Population stratification analyses supported the generalizability of these findings across diverse subgroups (all *P* for interaction > 0.05).

**Conclusion:**

The HALP score demonstrated a negative correlation with the prevalence of low muscle mass and its associated mortality, indicating its utility as a combined indicator for risk assessment.

## Introduction

With increasing life expectancy globally, the proportion of older adults within the population is steadily growing. In 2020, the global population of individuals aged 60 and older was 1 billion, and it is expected to rise to 2.1 billion by 2050 (1). The changing demographic landscape has elevated the importance of addressing health and life satisfaction among seniors on a global scale. Sarcopenia, a chronic condition linked to aging that involves the loss of muscle tissue, diminished strength, and impaired functionality, constitutes a serious challenge to the health of older individuals (2–4). Globally, the prevalence of sarcopenia is estimated at 5 to 10%, with rates ranging from 11 to 50% in those over the age of 80 (5). This condition not only impairs daily functioning and increases the risk of falls and fractures, but is also associated with a variety of serious health conditions such as osteoporosis, obstructive sleep apnea (OSA), prediabetes, stroke, cancer and death (6–12). Given its widespread impact and serious consequences, sarcopenia has become an urgent public health priority, highlighting the need for early identification of high-risk individuals to enable timely prevention and intervention (13, 14).

The onset and progression of sarcopenia are significantly influenced by inflammation (15). It has been demonstrated that patients with sarcopenia typically experience a chronic inflammatory state, which is closely associated with hallmark features such as muscle atrophy, decreased muscle strength, and impaired muscle function (16, 17). Moreover, nutritional status is critical in the management of sarcopenia. Malnutrition, especially insufficient protein intake, impairs muscle protein synthesis, a key process in the pathogenesis of sarcopenia (18). Therefore, biomarkers that simultaneously reflect nutritional and inflammatory status may be particularly useful for sarcopenia risk assessment. Recently, the HALP score, which integrates hemoglobin, albumin, lymphocyte, and platelet measurements, has emerged as an economical and straightforward diagnostic marker (19). Each component of the HALP score is closely related to muscle health. Hemoglobin supports oxygen delivery to muscles; albumin reflects nutritional and protein status; lymphocyte count indicates immune and inflammatory balance; and platelets are involved in systemic inflammation. Together, these markers represent key physiological pathways that influence muscle mass and function. Compared with other inflammation-related indices such as the neutrophil-to-lymphocyte ratio (NLR), systemic immune-inflammation index (SII), the HALP score reflects both nutritional and immune-inflammatory status in a single composite value (20–22). Moreover, all four components of HALP are routinely measured in standard blood tests and do not depend on C-reactive protein (CRP) or other specialized assays, thereby enhancing its practicality and ease of implementation in clinical settings. Initially introduced by Chen et al. in 2015 as a prognostic indicator for gastric cancer (23), the HALP score has since been recognized for its potential as a biomarker for various diseases. Research has established links between the HALP score and conditions such as chronic obstructive pulmonary disease, diabetic retinopathy, and erectile dysfunction (24–26). Furthermore, it exhibits a significant prognostic correlation with multiple types of cancer (27–32).

However, despite its widespread application in other diseases, the relationship between the HALP score and sarcopenia remains undefined. While the complete diagnosis of sarcopenia requires assessing both muscle mass and function, low muscle mass represents its core pathological feature and is a feasible outcome to assess in large-scale epidemiological studies. To bridge the current research gap, this study utilizes the National Health and Nutrition Examination Survey (NHANES) datasets to examine the association between the HALP score and the prevalence of low muscle mass, alongside its prognostic significance for all-cause mortality in individuals identified with this condition.

## Methods

### Study population

The NHANES survey employed a stratified, multi-stage random sampling methodology, ensuring that samples were drawn from a broad range of geographic regions and diverse population groups across the United States, thus enhancing the national representativeness of the results. Participants underwent extensive physical exams, completed detailed health and nutrition questionnaires, and provided laboratory specimens (33). These data offer invaluable and reliable multi-dimensional insights for research in health-related fields. The NHANES study protocol was approved by the Institutional Review Board of the National Center for Health Statistics, with all participants providing informed consent. Importantly, the NHANES dataset does not contain any personally identifiable sensitive information.

**Figure 1:**
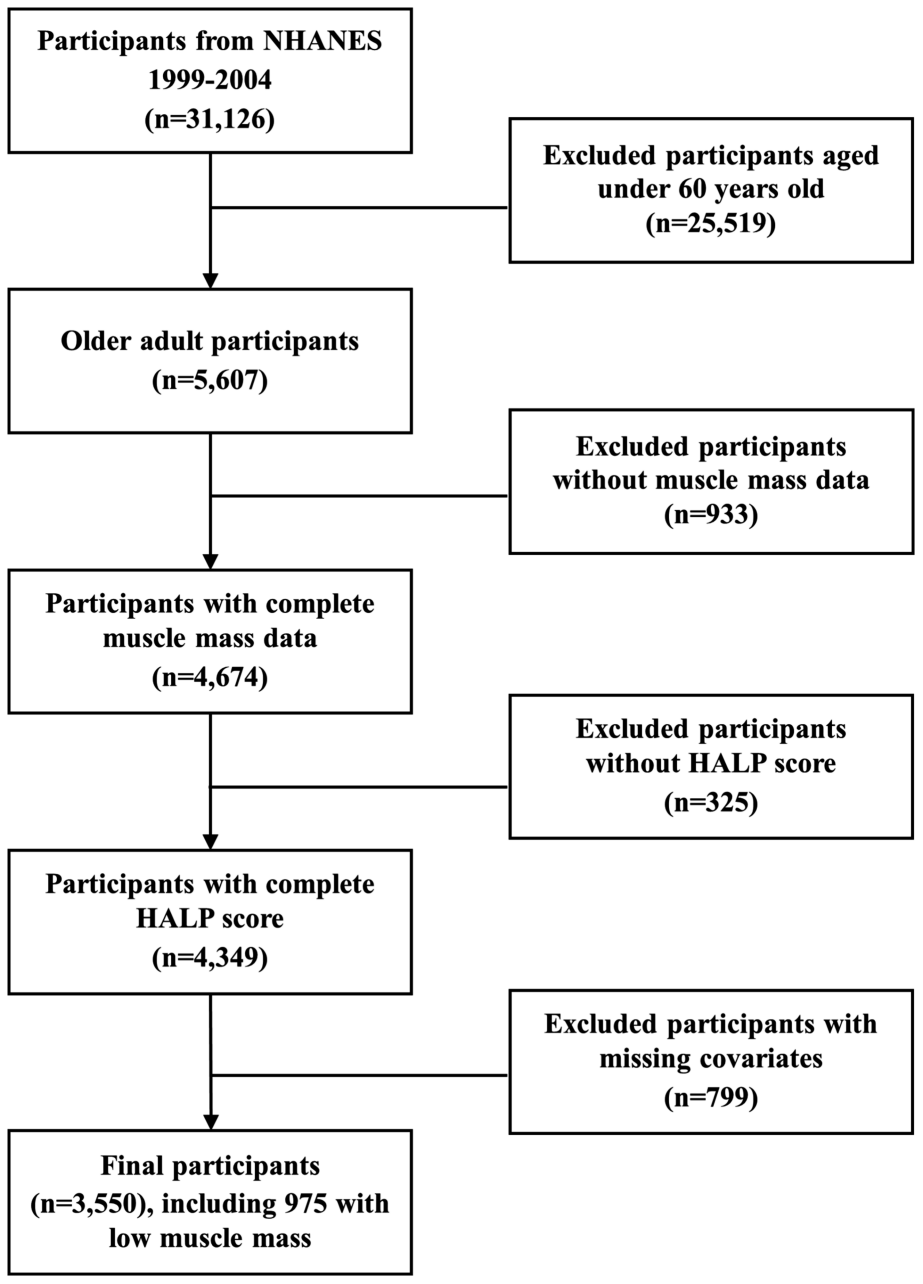
Flow chart of the study.

This study integrated data from three consecutive NHANES two-year cycles (1999–2004), involving a total of 31,126 participants. Based on predefined exclusion criteria, 25,519 participants under the age of 60, 933 participants without appendicular skeletal muscle mass data, 325 participants missing HALP score, and 799 participants excluded due to missing or abnormal covariate data were excluded. A total of 3,550 individuals were ultimately enrolled in the study. The flowchart illustrating the selection procedure is presented in Figure 1. Furthermore, among the 3,550 participants, 975 participants with low muscle mass were included in the subsequent survival analysis.

### Outcome assessment

The primary outcome of this study was low muscle mass, a key component of the sarcopenia syndrome. The presence of low muscle mass was determined based on the sex-specific criteria for the appendicular skeletal muscle mass index established by the Foundation for the National Institutes of Health (FNIH) Sarcopenia Project, with cutoff points set at 0.789 for males and 0.512 for females (34). These thresholds are widely used in epidemiological research (9, 35, 36). The muscle index is derived by dividing the mass of appendicular skeletal muscles (kg) by the body mass index (BMI, kg/m^2^) (37). Within the NHANES framework, appendicular skeletal muscle mass was assessed using dual-energy X-ray absorptiometry (DXA). It is important to clarify that this study focuses solely on the low muscle mass component, as comprehensive data on muscle strength or physical performance were unavailable to apply multi-component diagnostic criteria for sarcopenia.

The secondary outcome was all-cause mortality. Death status was determined through a probabilistic matching of NHANES data with public-use mortality files from the National Death Index (NDI), a standard procedure conducted by the National Center for Health Statistics (NCHS) to ensure high-accuracy follow-up. For the purpose of this all-cause mortality analysis, no participants were excluded due to uncertain or incomplete cause-of-death information, ensuring all ascertained deaths were included. The follow-up period starts from the date of the NHANES examination and continues until the date of death or December 31, 2019, whichever occurs first. Causes of death are classified according to the International Classification of Diseases, 10th edition (ICD-10). All-cause mortality refers to the total number of deaths from any cause during the specified follow-up period.

### Exposure variables

During the recruitment process, each participant provided a blood sample, which was processed and stored at −20°C before being sent to a certified laboratory for comprehensive analysis. Blood cell analysis was conducted with the Beckman Coulter MAXM system, and albumin concentrations in serum were measured via the Beckman Synchron LX20 analyzer. The HALP score was derived through the equation: hemoglobin concentration (g/L) multiplied by albumin level (g/L) multiplied by lymphocyte number (/L), divided by platelet count (/L) (38).

### Covariate definitions

We selected covariates based on prior literature and clinical relevance to sarcopenia or low muscle mass, including sociodemographic characteristics, lifestyle factors, and health status. NHANES collected data on participants’ gender, age, race, marital status, education level, poverty-to-income ratio (PIR), smoking and alcohol use, moderate activity, muscle strengthening activity, as well as medical histories of hypertension and diabetes through household interviews. In the mobile examination center, participants’ BMI was measured. Laboratory tests included alanine aminotransferase (ALT), creatinine (SCr), total calcium, and CRP levels. Diagnoses of smoking, alcohol use, hypertension, and diabetes were determined based on relevant questionnaire items (SMQ020, ALQ101, DIQ010, BPQ020). Moderate activity and muscle strengthening activity were assessed using questionnaire items PAD320 and PAD440, respectively.

### Statistical analysis

To address the significant right-skewed distribution observed in HALP score data, the natural logarithm (ln HALP) was utilized for transformation. This preprocessing step not only enhanced the fit of the statistical model but also improved the robustness of the analysis and the interpretability of the results.

Participants were categorized into quartiles based on the distribution of HALP score in the overall study population. Quartile cut-off values were calculated using the 25th, 50th, and 75th percentiles to ensure approximately equal sample sizes across groups. To ensure the study sample accurately reflects the national population, we made the necessary adjustments using sample weights (WTMEC2YR), stratification (SDMVSTRA), and clustering (SDMVPSU). Continuous variables were presented as mean ± standard deviation (SD), while categorical variables were presented as frequencies or percentages. The significance of intergroup differences was assessed using weighted chi-square tests and weighted rank sum tests.

To investigate the relationship between ln HALP and low muscle mass, multivariable logistic regression analysis was performed, with results presented as odds ratios (OR) and 95% confidence intervals (CI). To evaluate the association between ln HALP and all-cause mortality in participants with low muscle mass, we used the Cox proportional hazards model, with results expressed as hazard ratios (HR) and 95% CI. The ln HALP was evaluated as both continuous and categorical variables, using the first quartile (Q1) as the baseline. Three analytical models were developed: Model 1 represented the crude unadjusted analysis; Model 2 incorporated adjustments for gender, race, marital status, education level, PIR; and Model 3 included additional covariates such as BMI, smoking, alcohol use, diabetes, hypertension, moderate activity, muscle strengthening, CRP, ALT, SCr, and total calcium. Model 3, the fully adjusted model, was selected as the primary basis for interpretation due to its theoretical relevance and comprehensive control of known confounders.

To explore the dose–response relationship (including both linear and non-linear associations) between ln HALP and low muscle mass, as well as all-cause mortality in participants with low muscle mass, restricted cubic spline (RCS) analysis was performed, adjusting for potential confounders as outlined in the fully adjusted model, with the 5th, 50th, and 95th percentiles of ln HALP used as knot points. We also identified potential inflection points and conducted threshold effect analysis. Furthermore, survival probability plots using the Kaplan–Meier method were generated to assess overall mortality risk in individuals with low muscle mass, with stratification performed according to quartile divisions of ln HALP.

Following adjustments for covariates in the primary analysis model (Model 3), subgroup analyses were conducted by gender (male or female), race (Mexican American, other races, non-Hispanic White, and non-Hispanic Black), marital status (married or unmarried and others), education level (less than high school, high school or GED, above high school), BMI (<25 kg/m^2^, 25–30 kg/m^2^, >30 kg/m^2^), smoking (yes or no), and alcohol use (yes or no). Interaction tests were also performed to evaluate possible variations in relationships among different subgroups. Furthermore, Sensitivity analysis was performed. We extended the mortality risk analysis to the overall population and to the subgroup without low muscle mass. To assess whether the presence of low muscle mass modified the effect of ln HALP on all-cause mortality, we conducted a subgroup analysis stratified by this condition and performed an interaction test.

The R Studio platform was utilized for conducting all data processing and statistical assessments. Significance thresholds were established at *p* < 0.05 for two-sided tests.

## Results

### Baseline information

A total of 3,550 participants were included in this study, representing approximately 33,045,140 elderly individuals in the United States. Table 1 presents the baseline characteristics of all participants. The mean age was 70.75 ± 7.53 years, with 44.8% of participants being male and 82.9% non-Hispanic White. The mean ln HALP value was 3.81 ± 0.44, and the overall prevalence of low muscle mass was 22.4%. Further analysis indicated that participants in the top quartile (Q4) of ln HALP were more likely to be male, younger, married, smokers, have higher ALT levels, lower CRP levels, higher total calcium levels, and have a greater likelihood of diabetes compared to those in the bottom quartile (Q1).

**Table 1:** Participant baseline characteristics stratified by ln HALP quartiles.

Characteristics	Overall, *N* = 33,045,140 Overall, *n* = 3,550	Q1 *n* = 888	Q2 *n* = 887	Q3 *n* = 887	Q4 *n* = 888	*p*-value
Age (years)	70.75 ± 7.53	72.02 ± 7.73	70.43 ± 7.53	70.24 ± 7.14	70.12 ± 7.41	<0.001
Gender (%)						<0.001
Male	44.8%	36.4%	41.5%	48.5%	53.7%	
Female	55.2%	63.6%	58.5%	51.5%	46.3%	
Race (%)						0.002
Mexican American	3.1%	2.3%	2.6%	3.2%	4.5%	
Other Race	6.8%	5.3%	6.7%	5.9%	9.4%	
Non-Hispanic White	82.9%	83.6%	83.6%	84.7%	79.5%	
Non-Hispanic Black	7.1%	8.8%	7.0%	6.1%	6.6%	
Marital status (%)						0.044
Married	62.6%	57.5%	63.6%	64.7%	64.6%	
Unmarried and others	37.4%	42.5%	36.4%	35.3%	35.4%	
Education level (%)						0.085
Less than high school	28.2%	26.3%	25.5%	28.6%	32.7%	
High school or GED	29.2%	27.5%	31.1%	30.8%	27.1%	
Above high school	42.7%	46.2%	43.4%	40.5%	40.2%	
PIR (%)						0.453
<1.5	26.4%	26.8%	25.1%	24.5%	29.3%	
1.5–3.5	39.0%	40.5%	37.7%	40.4%	37.1%	
>3.5	34.6%	32.7%	37.1%	35.1%	33.6%	
BMI (kg/m^2^, %)						0.207
<25	29.3%	33.1%	29.8%	27.5%	26.6%	
25–30	39.7%	37.6%	41.4%	39.0%	40.9%	
>30	30.9%	29.2%	28.8%	33.5%	32.5%	
Smoking (%)						0.019
Yes	53.9%	52.8%	50.0%	55.5%	57.8%	
No	46.1%	47.2%	50.0%	44.5%	42.2%	
Alcohol use (%)						0.460
Yes	60.1%	57.9%	60.7%	59.7%	62.3%	
No	39.9%	42.1%	39.3%	40.3%	37.7%	
Moderate activity (%)						0.560
Yes	46.3%	45.1%	46.9%	48.6%	44.4%	
No	53.7%	54.9%	53.1%	51.4%	55.6%	
Muscle strengthening (%)						0.804
Yes	16.5%	15.6%	17.0%	17.5%	15.8%	
No	83.5%	84.4%	83.0%	82.5%	84.2%	
Diabetes (%)						0.012
Yes	15.6%	14.6%	12.5%	17.0%	18.5%	
No	84.4%	85.4%	87.5%	83.0%	81.5%	
Hypertension (%)						0.066
Yes	53.0%	57.1%	50.9%	52.0%	51.9%	
No	47.0%	42.9%	49.1%	48.0%	48.1%	
Low muscle mass (%)						0.176
Yes	22.4%	25.2%	20.9%	20.2%	23.2%	
No	77.6%	74.8%	79.1%	79.8%	76.8%	
CRP (mg/dL)	0.51 ± 0.94	0.70 ± 1.31	0.47 ± 0.87	0.43 ± 0.67	0.42 ± 0.74	<0.001
ALT (U/L)	22.39 ± 10.77	20.36 ± 9.04	21.36 ± 9.44	22.50 ± 10.57	25.65 ± 13.12	<0.001
SCr (μmol/L)	83.99 ± 40.40	89.12 ± 53.91	83.24 ± 41.62	81.39 ± 30.25	82.01 ± 29.41	0.119
Total calcium (mmol/L)	2.38 ± 0.10	2.36 ± 0.11	2.37 ± 0.10	2.39 ± 0.11	2.39 ± 0.10	<0.001

Continuous variables were showed as mean ± SD, categorical variables were showed as percentage.

PIR, Poverty Index Ratio; BMI, Body Mass Index; CRP, C-Reactive Protein; ALT, Alanine Aminotransferase; SCr, Serum Creatinine.

### Relationship between the ln HALP and low muscle mass

Table 2 presents the results of the multivariable logistic regression analysis investigating the relationship between ln HALP and low muscle mass. When ln HALP was analyzed as a continuous variable, the results from model 2 showed an OR of 0.76 (95% CI: 0.64, 0.90), indicating a negative association. This inverse relationship remained significant in the fully adjusted model (OR = 0.72, 95% CI: 0.60, 0.87). Further stratification of ln HALP into quartiles showed that, compared to the Q1 group, participants in the Q4 group had a lower risk of low muscle mass in the fully adjusted model. Specifically, compared to participants in the bottom quartile (Q1), the risk of low muscle mass in the top quartile (Q4) was reduced by 29% (OR = 0.71, 95% CI: 0.56, 0.89, *P* for trend < 0.05).

**Table 2:** Association between ln HALP and low muscle mass.

Characteristic	Model 1			Model 2			Model 3		
	OR	95% CI	*P*-value	OR	95% CI	*P*-value	OR	95% CI	*P*-value
ln HALP (continuous)	1.00	0.85, 1.17	0.969	0.76	0.64, 0.90	0.002	0.72	0.60, 0.87	<0.001
ln HALP									
Q1	Ref	Ref		Ref	Ref		Ref	Ref	
Q2	0.88	0.71, 1.08	0.227	0.78	0.63, 0.98	0.029	0.77	0.61, 0.97	0.026
Q3	0.84	0.68, 1.04	0.112	0.66	0.53, 0.82	<0.001	0.62	0.49, 0.78	<0.001
Q4	1.04	0.85, 1.28	0.676	0.75	0.60, 0.93	0.010	0.71	0.56, 0.89	0.004
*P* for trend			0.775			0.004			0.001

Model 1: Unadjusted.

Model 2: Adjusted for Gender and Race, Marital status, Education level, PIR.

Model 3: Adjusted for Gender, Race, Marital status, Education level, PIR, BMI, Smoking, Alcohol use, Moderate activity, Muscle strengthening, Diabetes, Hypertension, CRP, ALT, SCr, Total calcium.

We constructed three Cox regression models to evaluate the independent association between ln HALP levels and all-cause mortality in participants with low muscle mass (Table 3). From the crude model to the fully adjusted model, the HR and 95% CI were 0.69 (0.58, 0.81), 0.71 (0.60, 0.84), and 0.76 (0.64, 0.91), respectively, with all models showing statistical significance (*p* < 0.05). When ln HALP was stratified into quartiles, the Q4 group showed a 23% lower risk of all-cause mortality compared to the Q1 group (HR = 0.77, 95% CI: 0.62, 0.97, *P* for trend < 0.05).

**Table 3:** Associations between ln HALP and all-cause mortality in participants with low muscle mass.

Characteristic	Model 1			Model 2			Model 3		
	HR	95% CI	*P*-value	HR	95% CI	*P*-value	HR	95% CI	*P*-value
ln HALP (continuous)	0.69	0.58, 0.81	<0.001	0.71	0.60, 0.84	<0.001	0.76	0.64, 0.91	0.003
ln HALP									
Q1	Ref	Ref		Ref	Ref		Ref	Ref	
Q2	0.68	0.55, 0.84	<0.001	0.70	0.57, 0.86	<0.001	0.70	0.57, 0.86	<0.001
Q3	0.62	0.51, 0.77	<0.001	0.64	0.52, 0.80	<0.001	0.66	0.53, 0.82	<0.001
Q4	0.65	0.53, 0.80	<0.001	0.69	0.55, 0.86	<0.001	0.77	0.62, 0.97	0.026
*P* for trend			<0.001			<0.001			0.017

Model 1: Unadjusted.

Model 2: Adjusted for Gender and Race, Marital status, Education level, PIR.

Model 3: Adjusted for Gender, Race, Marital status, Education level, PIR, BMI, Smoking, Alcohol use, Moderate activity, Muscle strengthening, Diabetes, Hypertension, CRP, ALT, SCr, Total calcium.

**Figure 2:**
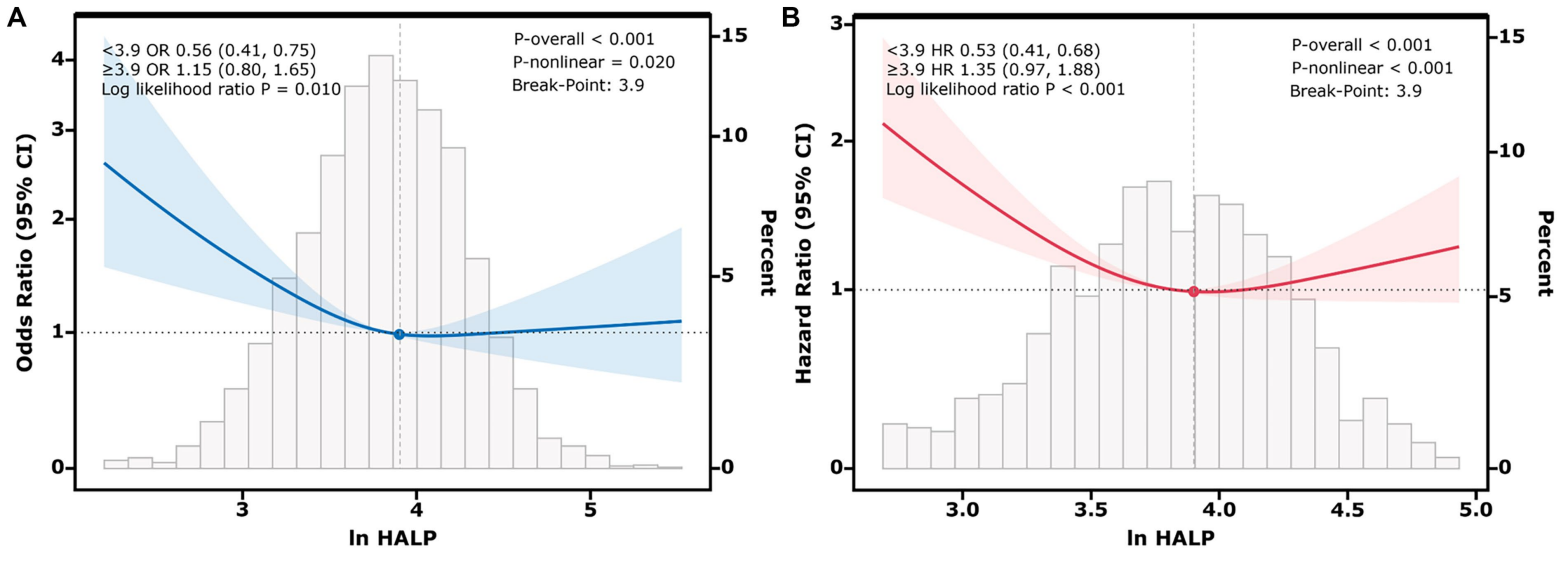
Restricted cubic spline (RCS) plots showing the nonlinear relationship between ln HALP and low muscle mass **(A)**, and between ln HALP and all-cause mortality in participants with low muscle mass **(B)**. The *Y*-axis represents the Odds Ratio (OR) in **(A)** and the Hazard Ratio (HR) in **(B)**. The solid line represents the adjusted association, and the shaded area represents the 95% confidence interval. The analysis identifies an inflection point at ln HALP = 3.9, indicated by the vertical dashed line. This threshold suggests that below this value, a higher HALP score is strongly associated with lower risk, whereas above this value, the protective association plateaus. The histogram at the bottom illustrates the distribution of ln HALP values among participants.

Additionally, the RCS analysis revealed a non-linear relationship between ln HALP and both the prevalence of low muscle mass and all-cause mortality in participants with low muscle mass (*P*-non-linear < 0.05). The threshold effect analysis presented in Figure 2A identified a critical value at ln HALP = 3.9. When ln HALP was less than 3.9 (OR = 0.56, 95% CI: 0.41, 0.75), ln HALP was negatively correlated with the prevalence of low muscle mass; however, no significant difference was observed when ln HALP was greater than or equal to 3.9 (OR = 1.15, 95% CI: 0.80, 1.65). Similarly, the threshold effect analysis in Figure 2B also identified a cutoff at ln HALP = 3.9. When ln HALP was less than 3.9 (HR = 0.53, 95% CI: 0.41, 0.68), higher ln HALP levels were significantly associated with reduced all-cause mortality in participants with low muscle mass. However, when ln HALP was greater than or equal to 3.9 (HR = 1.35, 95% CI: 0.97, 1.88), no significant statistical difference was observed.

**Figure 3:**
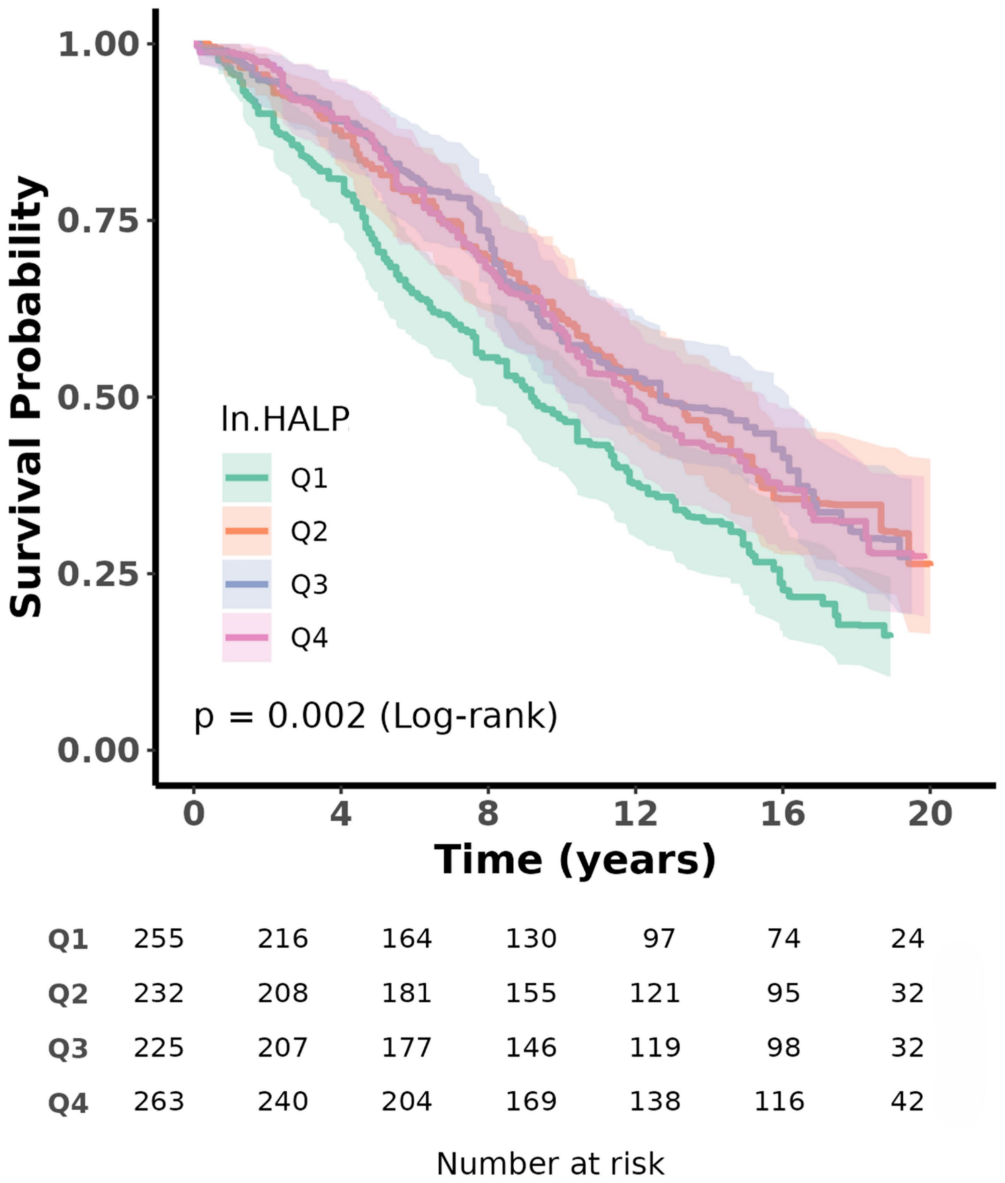
Kaplan–Meier survival analysis plot for all-cause mortality with quartile groups of ln HALP.

The Kaplan–Meier survival analysis showed a significant overall difference in survival probabilities across the four ln HALP quartiles (*p* < 0.05, log-rank) (Figure 3). Participants in the highest quartile (Q4) had a visibly higher long-term survival rate compared to those in the lowest quartile (Q1).

### Subgroup analyses

**Figure 4:**
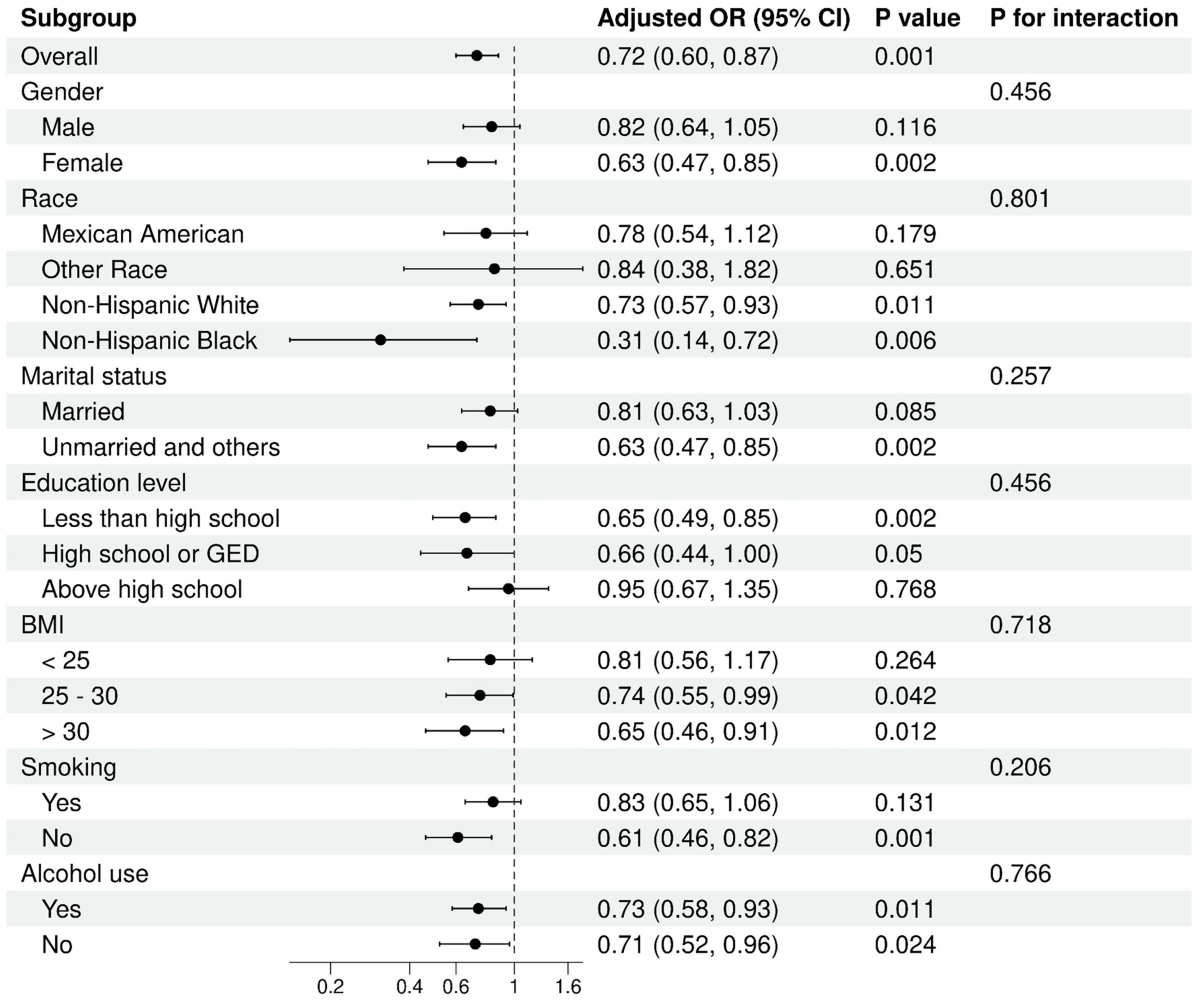
Subgroup analysis of ln HALP with low muscle mass.

**Figure 5:**
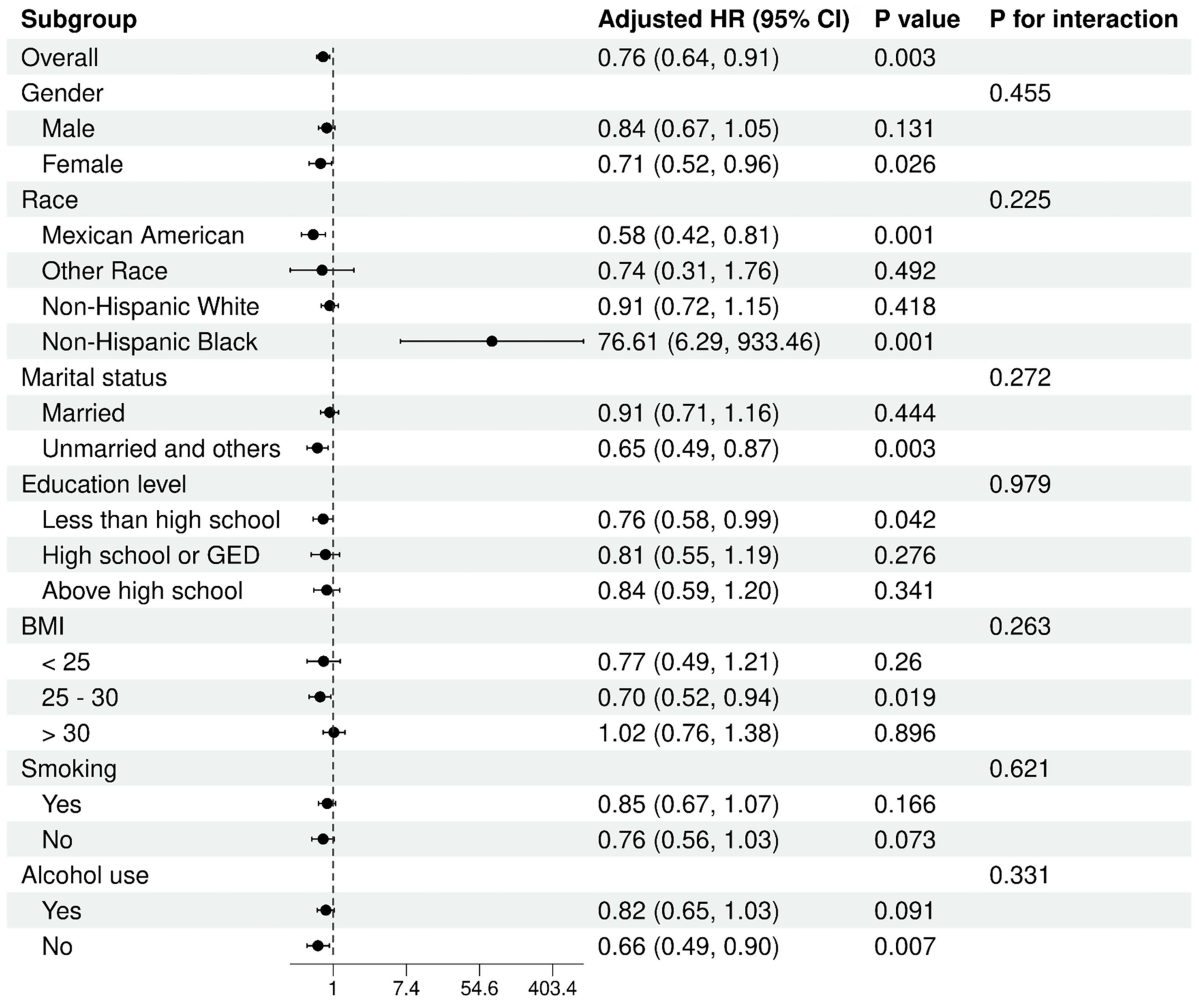
Subgroup analysis of ln HALP with all-cause mortality in participants with low muscle mass.

Subgroup analyses indicated no meaningful variations across groups (all *P* for interaction > 0.05), reinforcing the reliability and consistency of these findings across different populations (Figures 4, 5). In the analysis of low muscle mass, stronger inverse associations were observed in females (OR = 0.63, 95% CI: 0.47, 0.85) and in participants with a BMI > 30 (OR = 0.65, 95% CI: 0.46, 0.91). Similarly, for all-cause mortality, the association between ln HALP and reduced risk appeared more prominent in females (HR = 0.71, 95% CI: 0.52, 0.96) and in those with a BMI between 25 and 30 (HR = 0.70, 95% CI: 0.52, 0.94).

### Sensitivity analyses

Sensitivity analyses conducted in the overall population (Supplementary Table S1) and in the subgroup without low muscle mass (Supplementary Table S2) showed that ln HALP remained negatively associated with all-cause mortality after multivariable adjustment, thereby reinforcing the robustness and generalizability of our primary findings. To specifically test for potential effect modification, we performed an interaction test to formally assess whether the effect of ln HALP on all-cause mortality differed between participants with and without low muscle mass. The analysis, conducted in the total population, revealed no statistically significant interaction (*P* for interaction > 0.05) (Supplementary Table S3). This suggests that the protective association of a higher HALP score on survival is consistent across both groups.

## Discussion

In this study, 3,550 participants were included, revealing a notable negative correlation between HALP score and low muscle mass prevalence and mortality. Notably, these relationships exhibited a non-linear pattern with a threshold at ln HALP = 3.9. Subgroup analysis and interaction tests further confirmed that stratifying variables did not significantly modify these associations, thus affirming the robustness of the results. The results underscore the utility of the HALP score as an accessible and reliable indicator for low muscle mass in the elderly population, offering new insights into the assessment of survival risks associated with this condition. Currently, there is a lack of research specifically investigating the relationship between HALP score and the prevalence and mortality of low muscle mass in older adults, thereby providing a novel research avenue for the field.

Multiple studies have demonstrated strong associations between inflammatory markers, nutritional indicators, and sarcopenia. A Turkish cross-sectional study of 105 older adults identified the NLR as an independent predictor of sarcopenia (OR = 1.31, 95% CI: 1.06, 1.62) (39). Similarly, Shi et al. (40) analyzed NHANES datasets and identified a notable correlation between the SII and reduced muscle mass. Their analysis showed that individuals in the top SII quartile (Q4) had a 28% higher chance of developing low muscle mass compared to those in the bottom quartile (Q1) (OR = 1.28, 95% CI: 1.16, 1.40). Guo et al.’s (20) recent study further supports this, revealing that complete blood count (CBC)-derived inflammatory markers are associated with both sarcopenia prevalence and mortality. Regarding nutritional factors, UK Biobank data indicate that sarcopenia is closely linked to older age, inflammatory status, and reduced serum albumin levels (41). Atteveld et al. (42), through a cross-sectional study of the Amsterdam Geriatrics Center cohort, confirmed that low albumin levels are significantly associated with decreased walking speed (*β* = −0.020, 95% CI: −0.028, −0.011) and reduced grip strength (β = −0.596, 95% CI: −0.881, −0.311). In another study, Li et al. (43) merged NHANES information with data from Kunshan Hospital in China, demonstrating that the C-reactive protein-albumin-lymphocyte (CALLY) index exhibited a negative correlation with sarcopenia rates in older and middle-aged adults. These findings were consistent across US community populations (OR = 0.26, 95% CI: 0.11, 0.56) and Chinese hospital patients (OR = 0.35, 95% CI: 0.12, 0.96) (43). Collectively, the outcomes of these studies are consistent with our findings regarding the HALP score and low muscle mass, highlighting the combined influence of inflammatory processes and nutritional health on the onset and advancement of sarcopenia, and providing a compelling rationale for developing multidimensional biomarker-based screening and intervention strategies.

The increasing prevalence of sarcopenia and its related mortality rates have emerged as significant health challenges in older populations, influenced by multiple interconnected pathophysiological processes (44, 45). Key contributors to its onset and progression include advanced age, inadequate nutrition, oxidative damage, impaired mitochondrial activity, and persistent inflammatory responses (46). The HALP score, as a composite index reflecting hemoglobin, albumin, lymphocyte, and platelet levels, may capture the complex interplay of these factors. Hemoglobin plays a central role in maintaining muscle health by facilitating oxygen delivery, modulating inflammatory responses, and supporting mitochondrial function, all of which are crucial for preserving muscle metabolism and function (47–49). Serum albumin, a key protein synthesized by the liver, has a multifaceted role in muscle health (50). It serves multiple functions, acting as a reservoir for amino acids for muscle protein synthesis, transporting hormones, and functioning as a major circulating antioxidant that scavenges free radicals (51). However, under conditions of high oxidative stress, such as those that occur with aging, albumin itself can become oxidized. This process not only impairs its protective antioxidant functions but also serves as an indicator of systemic oxidative damage, a state which is thought to contribute to the gradual decline in muscle mass (52). Specific subsets of lymphocytes, such as CD4 + CD28 null T cells, have been found to be negatively correlated with muscle mass, suggesting their involvement in muscle degradation in sarcopenia (53). Myokines, muscle-derived cytokines, further highlight the connection between immune function and muscle health by modulating lymphocyte activity (54). Platelets, essential for coagulation, also play significant roles in regulating inflammation and oxidative stress (55). Through the release of inflammatory mediators such as platelet factor 4 (PF4), platelets initiate vascular inflammatory responses and remodeling, disrupting microcirculatory function and damaging microvascular endothelium. This disruption can lead to an imbalance in the supply of oxygen and nutrients to muscle tissues, thereby exacerbating muscle metabolic abnormalities (56, 57). Taken together, these mechanistic insights underscore the rationale for using composite indices that integrate multiple physiological domains. Our study provides empirical evidence for this association and supports the potential utility of HALP as a pragmatic, integrative biomarker for identifying individuals at elevated risk of low muscle mass and mortality in aging populations. Future research should focus on longitudinal validation and mechanistic elucidation to advance HALP from an observational marker to a clinically actionable tool.

Notably, our study identified a non-linear association with a critical threshold at ln HALP = 3.9, beyond which the inverse relationship between the HALP score and both low muscle mass and all-cause mortality began to plateau. This phenomenon may have both pathophysiological and clinical explanations. From a pathophysiological perspective, this threshold may represent a point where an individual’s basic nutritional and inflammatory status is no longer the primary limiting factor for muscle health. When the HALP score is below 3.9, it likely reflects a state of significant nutritional deficiency and/or chronic inflammation, which directly impairs muscle protein synthesis and promotes catabolism (15). In this range, improving nutritional-inflammatory status can yield substantial benefits. However, once the HALP score exceeds this threshold, it suggests a “ceiling effect” has been reached where nutritional and immune functions are relatively adequate. At this stage, other, potentially irreversible age-related factors may become the dominant drivers of muscle loss (6). From a clinical standpoint, these findings are highly relevant. The HALP score, derived from routine and inexpensive blood tests, can serve as a practical and cost-effective screening tool. The inflection points at 3.9 is particularly crucial for risk stratification and guiding targeted interventions. For elderly individuals with an ln HALP score below 3.9, they represent a high-risk population that may benefit most from nutritional supplementation and anti-inflammatory therapies (18). Conversely, for individuals with a score above 3.9, whose nutritional-inflammatory status is relatively adequate, the clinical focus should shift toward other key interventions, such as structured physical exercise programs, to maintain muscle health (13). By integrating markers of both nutrition and inflammation, the HALP score offers a more holistic view of an individual’s physiological status, paving the way for more personalized preventive strategies against muscle loss in older adults.

Although the crude prevalence of low muscle mass did not exhibit a clear linear trend across HALP quartiles, and some higher HALP groups showed increased rates of diabetes and smoking, the multivariable logistic regression analysis revealed a consistent inverse association between HALP levels and low muscle mass risk. This apparent discrepancy may be explained by baseline confounding factors such as sex, BMI, diabetes, and smoking, which were unevenly distributed across quartiles. After adjusting for these variables, the protective association of HALP with both low muscle mass and all-cause mortality remained statistically significant. These findings suggest that HALP independently reflects an underlying nutritional-inflammatory status that may mitigate the adverse effects of traditional risk factors. Importantly, our interaction analysis demonstrated that this protective effect on mortality was consistent regardless of the presence of low muscle mass, strengthening its role as a general prognostic biomarker in the older population. Furthermore, Stratified results indicated that the inverse association between HALP and both low muscle mass and all-cause mortality was more pronounced among females and participants with higher BMI. These observations may reflect inherent sex-based or metabolic differences influencing inflammation, nutritional reserves, or muscle metabolism. Although exploratory, these trends may carry clinical implications and deserve further investigation in targeted populations. Sensitivity analyses confirmed that the negative association between ln HALP and all-cause mortality was robust across different population subsets, supporting the stability and generalizability of the main findings. These results suggest that HALP score is a valuable and pragmatic biomarker, especially in resource-limited contexts where simplicity, accessibility, and reproducibility are essential.

This study has several notable strengths. First, the large sample analysis based on the NHANES dataset, combined with sample design and weight adjustments, enhances the national representativeness of the results. Second, the study carefully considered multiple potential confounders, ensuring the robustness of the conclusions. Furthermore, subgroup analyses across different populations further validated the consistency of the results. However, the study also has some limitations. First, although we adjusted for multiple covariates, the possibility of residual confounding remains. For instance, we could not account for specific comorbidities or medication use that can independently influence HALP components, nor for detailed dietary patterns, which could impact the results. Second, the HALP score was calculated based on a single time-point measurement. Given that nutritional and inflammatory statuses are dynamic variables, this single snapshot may not fully represent an individual’s long-term condition. This could introduce temporal misclassification bias and potentially underestimate the true strength of the observed associations. Third, since the NHANES cohort was designed to represent the U. S. civilian population, the generalizability of these findings to other populations, particularly Asian elderly cohorts, requires further validation in independent and ethnically diverse datasets.

## Conclusion

To summarize, our research reveals a strong inverse relationship between the HALP score and both the prevalence of low muscle mass and all-cause mortality in those with the condition among older adults. However, to establish the HALP score as a reliable diagnostic and prognostic tool, further validation through well-constructed prospective cohort studies across various populations is essential.

## Data Availability

Publicly available datasets were analyzed in this study. This data can be found: www.cdc.gov/nchs/NHANEs/.
