# Supplementary material for: The association of HALP score with low muscle mass in older adults
Source: Front Nutr. 2025 Aug 19;12:1618736. doi: 10.3389/fnut.2025.1618736 (PMC12401707; doi:10.3389/fnut.2025.1618736)
Supplement: Supplementary file 1 [file Table_1.docx]

**Table S1 Associations between ln HALP and all-cause mortality in all participants**

| **Characteristic** | **Model 1** | | | **Model 2** | | | **Model 3** | | |
| --- | --- | --- | --- | --- | --- | --- | --- | --- | --- |
|  | **HR** | **95% CI** | ***P*-value** | **HR** | **95% CI** | ***P*-value** | **HR** | **95% CI** | ***P*-value** |
| **ln HALP (continuous)** | 0.75 | 0.68, 0.82 | <0.001 | 0.72 | 0.65, 0.79 | <0.001 | 0.78 | 0.70, 0.86 | <0.001 |
| **ln HALP** |  |  |  |  |  |  |  |  |  |
| **Q1** | Ref | Ref |  | Ref | Ref |  | Ref | Ref |  |
| **Q2** | 0.77 | 0.68, 0.86 | <0.001 | 0.78 | 0.70, 0.88 | <0.001 | 0.81 | 0.72, 0.91 | <0.001 |
| **Q3** | 0.72 | 0.64, 0.81 | <0.001 | 0.71 | 0.63, 0.80 | <0.001 | 0.73 | 0.65, 0.82 | <0.001 |
| **Q4** | 0.74 | 0.66, 0.83 | <0.001 | 0.71 | 0.63, 0.80 | <0.001 | 0.77 | 0.68, 0.87 | <0.001 |
| **P for trend** |  |  | <0.001 |  |  | <0.001 |  |  | <0.001 |
| **Model 1: Unadjusted Model 2: Adjusted for Gender and Race, Marital status, Education level, PIR Model 3: Adjusted for Gender, Race, Marital status, Education level, PIR, BMI, Smoking, Alcohol use, Moderate activity, Muscle strengthening, Diabetes, Hypertension, CRP, ALT, SCr, Total calcium** | | | | | | | | | |
