# Supplementary material for: The association of HALP score with low muscle mass in older adults
Source: Front Nutr. 2025 Aug 19;12:1618736. doi: 10.3389/fnut.2025.1618736 (PMC12401707; doi:10.3389/fnut.2025.1618736)
Supplement: Supplementary file 3 [file Table_3.docx]

**Table S3 Subgroup analysis of ln HALP with all-cause mortality in all participants**

| **Subgroup** | **Adjusted HR (95% CI)** | ***P*-value** | ***P* for interaction** |
| --- | --- | --- | --- |
| **Overall** | 0.78 (0.70, 0.86) | <0.001 |  |
| **Low muscle mass** |  |  | 0.33 |
| **Yes** | 0.76 (0.64, 0.91) | 0.003 |  |
| **No** | 0.83 (0.74, 0.93) | 0.002 |  |
